# Optimizing MRI sequence selection for glioma classification: a radiomics-based analysis of WHO CNS grade, final pathological diagnosis, and IDH status

**DOI:** 10.1007/s11547-026-02218-9

**Published:** 2026-09-06

**Authors:** Kimberly Amador, Helge Kniep, Jens Fiehler, Nils Daniel Forkert, Thomas Lindner

**Affiliations:** 1 Department of Radiology and Clinical Neurosciences, University of Calgary, Calgary, Canada; 2 Department of Diagnostic and Interventional Neuroradiology, University Hospital Hamburg-Eppendorf, Martinistr. 52, 20251 Hamburg, Germany

**Keywords:** Radiomics, Machine Learning, Glioma

## Abstract

**Background:**

Multi-sequence MRI protocols for glioma classification are resource-intensive, prompting the need to identify optimal, potentially reduced imaging protocols.

**Purpose:**

To evaluate the diagnostic utility of individual MRI sequences (ASL, DWI, FLAIR, SWI, T1w, T2w) for classifying WHO CNS grade (II, III, IV), final pathological diagnosis (glioblastoma vs. astrocytoma), and IDH status (mutated, wildtype, IDH1) using radiomic features and machine learning.

**Materials and methods:**

We analyzed data from 501 patients with diagnosed brain cancer from the UCSF-PDGM dataset, by extracting radiomics features from all available imaging sequences and training uni- and multi-sequence machine learning models (ASL, DWI, FLAIR, SWI, T1w, T2w) for the three outcome tasks of interest. Performance metrics (accuracy, sensitivity, F1-score) were derived for each task, alongside feature selection frequencies.

**Results:**

The uni-sequence machine learning models using FLAIR imaging features excelled for WHO CNS grade classification (57.4% accuracy), ASL for final pathological diagnosis (81.5% accuracy), and T1w for IDH status classification (66.7% accuracy). Interestingly, the combination of all sequences did not lead to significant improvements compared to the best performing uni-sequence machine learning models for the three tasks. Regardless of the input sequence, the models consistently relied on similar features, such as shape and demographic variables (*e.g.,* patient age and tumor sphericity).

**Conclusion:**

Single sequences, such as FLAIR, ASL, and T1w, achieved the highest individual accuracies in the radiomics models for classification of tumor grade, final diagnosis, and IDH status, and may inform the design of more streamlined imaging protocols.

**Supplementary Information:**

The online version contains supplementary material available at https://doi.org/10.1007/s11547-026-02218-9.

## Introduction

Gliomas are the most common primary brain tumors, representing approximately 30% of all brain and central nervous system tumors and 80% of malignant brain tumors [1]. Accurate classification of gliomas is critical for patient management, as it directly influences prognosis and treatment decisions. The World Health Organization (WHO) classification system, which integrates histological and molecular features, has emerged as the gold standard for glioma diagnosis [2]. However, achieving a precise and non-invasive classification remains complex due to tumor heterogeneity and the lack of consensus on the optimal imaging sequences for multi-sequence MRI protocols used for clinical assessments.

Current MRI protocols for glioma imaging typically include a broad array of sequences that can differ vastly between centers. Commonly used sequences include T1-weighted imaging, T2-weighted imaging, fluid-attenuated inversion recovery (FLAIR), diffusion-weighted imaging (DWI), susceptibility-weighted imaging (SWI), and perfusion imaging like dynamic susceptibility contrast (DSC) or arterial spin labeling (ASL) [3]. While these sequences can provide detailed anatomical and functional insights about patient-individual brain tumors with their respective strengths, the comprehensive nature of a full MRI protocol comes at the cost of being resource-intensive and requiring long scan times, placing a burden on patients, particularly those with conditions that make them more prone to movement during the scanning procedure. In general, more comprehensive imaging data may support improved tumor classification. However, it remains uncertain whether all these sequences are truly essential and informative for an accurate classification or if a more specific subset of sequences could achieve comparable results for specific diagnostic tasks. Some of the most important tasks for current clinical applications are WHO CNS grading, the differential diagnosis between glioblastomas and astrocytomas, and profiling molecular markers, like isocitrate dehydrogenase (IDH) mutation status.

Recent advances in radiomics and machine learning offer novel opportunities to address these challenges [4, 5]. Radiomics enables the automated extraction of quantitative and semi-quantitative features from medical images, helping to reduce reliance on subjective image interpretation. These features can then be used to train machine learning models that identify patterns associated with clinical outcomes. By applying these techniques to MRI data, it becomes possible to systematically evaluate the diagnostic contribution of individual sequences and identify optimal combinations that are most informative for specific diagnostic questions [6]. However, comprehensive comparisons of MRI sequence-specific performance across multiple glioma classification tasks of clinical interest remain limited [7–9], leaving a gap in the evidence needed to guide clinical practice.

To address this gap, we leverage the UCSF-Preoperative Diffuse Glioma MRI (PDGM) dataset, which includes multi-sequence MRI data from 501 patients with diffuse glioma, encompassing ASL, DWI, FLAIR, SWI, T1-weighted, and T2-weighted MRI sequences, all of which are commonly used in clinical routine [10]. We hypothesize that, despite the complementary information captured by different imaging contrasts, a subset of sequences may provide sufficient discriminative signal for the various glioma classification tasks. If so, such targeted selection could improve efficiency by reducing redundant imaging acquisition, enabling shorter scan times, and improving patient comfort. Accordingly, the main objectives of this work are (1) to compare the performance of individual and combined MRI sequences for each classification task using radiomics-based machine learning models, (2) to identify the most predictive radiomic features across tasks, and (3) to propose more efficient MRI protocols that balance diagnostic precision with practical feasibility, *i.e.,* the reduction of the total number of sequences used. The three key classification tasks included in this study are: (1) WHO CNS grading, (2) differentiating glioblastoma from astrocytoma, and (3) determining the IDH mutation status.

## Materials and methods

### Dataset

In this study, the University of California, San Francisco-Pediatric Diffuse Glioma MRI (UCSF-PDGM) dataset, a publicly available single-institution retrospective cohort, was used. The dataset encompasses de-identified clinical and preoperative MRI data from 501 patients with diffuse gliomas, including 55 (11%) grade II, 42 (9%) grade III, and 403 (80%) grade IV tumors, with a male predominance (56–60% overall). Molecular markers available include IDH mutations (83% in grade II, 67% in grade III, 8% in grade IV), methylguanine-methyltransferase (MGMT) promoter hypermethylation (63% grade IV), and 1p/19q codeletion (20% grade II, 5% grade III, < 1% grade IV). WHO CNS tumor grade, final pathological diagnosis (glioblastoma vs. astrocytoma), and IDH mutation status were used as provided in the UCSF-PDGM dataset and served as reference standards for analysis.

A total of 15 patients had to be excluded due to insufficient subgroup representation: 13 with oligodendroglioma and two with IDH2 mutations. Thus, the final analysis was performed using data from 486 patients. Imaging was performed on a 3.0 Tesla scanner (Discovery 750, GE Healthcare). Image sequences available for all patients included: Pre- and post-contrast T1-weighted imaging, T2-weighted MRI, FLAIR, SWI, DWI, and ASL. Details regarding the imaging parameters have been described previously [11].

### Image preprocessing

Preprocessing involved various correction and enhancement procedures, including eddy current correction for the DTI data, and non-linear co-registration of all sequences to the FLAIR sequence, which served as the reference standard. Each individual FLAIR image was also aligned to the Montreal Neurological Institute (MNI152) atlas using translation-only registration. The image data first underwent automated segmentation using an ensemble model consisting of prior BraTS challenge-winning segmentation algorithms. Then, the images were manually corrected by trained radiologists and approved by two expert reviewers. The final segmentation included three major tumor compartments, namely enhancing tumor (contrast agent uptake), non-enhancing (necrotic) tumor, and surrounding FLAIR abnormality (edema). More details about the image preprocessing steps can be found in a previous publication [12].

### Radiomics feature extraction

Radiomics features were extracted from each MRI sequence and patient using the PyRadiomics library (version 3.0.1), an open-source Python package that follows the Image Biomarker Standardization Initiative (IBSI) guidelines to ensure standardized and reproducible imaging feature extraction. The standard settings were applied, encompassing a comprehensive set of feature classes, including first-order statistics (*e.g.,* mean, median, skewness), shape descriptors (*e.g.,* volume, surface area), and texture features, such as Gray Level Co-occurrence Matrix (GLCM), Gray Level Run Length Matrix (GLRLM), and Gray Level Size Zone Matrix (GLSZM). These features were calculated from both the original images and wavelet-decomposed transformations to capture multi-scale spatial information. The regions of interest (ROIs) were already predefined. As described above, the dataset provides ROIs for the enhancing tumor, non-enhancing/necrotic tumor, and surrounding FLAIR abnormality (*i.e.,* edema). In this study, the enhancing tumor ROIs were used. The extracted radiomics features then served as input for the subsequent AI-based classification task.

### Handling class imbalance

All outcome variables exhibited notable class imbalances. For tumor grade, 82% of cases were grade IV (*n* = 393), compared to approximately 9% each for grade II (*n* = 45) and grade III (*n* = 41). In the pathological diagnosis task, glioblastoma accounted for 76% (*n* = 366) and astrocytoma for 24% (*n* = 113) of all cases. For IDH genotype, 80% of patients were classified as wildtype (*n* = 384), 13% as IDH1 (*n* = 64), < 1% as IDH2 (*n* = 2) and 6% as mutant (*n* = 31). This imbalance can bias any downstream machine learning model toward the majority classes, limiting generalizability and reducing performance on minority classes [13].

To mitigate this problem, we employed a repeated random under-sampling strategy, generating 20 balanced datasets for each classification task. In each dataset, samples from the majority class(es) were randomly downsampled to match the size of the smallest class, thereby ensuring parity between class sizes while retaining representative diversity. Although under-sampling reduces the effective training set size and may increase estimator variance, repeating the procedure across multiple independent balanced datasets helps to mitigate dependence on any single reduced training set and supports robust performance estimation. The resulting balanced datasets were then used as input to the machine learning pipeline described below.

### Machine learning pipeline

We conducted three experiments using an in-house machine learning pipeline to predict the following clinical outcomes: (1) WHO CNS tumor grade (II, III, or IV), (2) final pathological diagnosis (glioblastoma or astrocytoma), and (3) IDH genotype (wildtype, mutant, or IDH1). Each outcome was modeled independently using the radiomics features extracted from a single MRI sequence (ASL, DWI, FLAIR, SWI, T1w, or T2w), combined with patient demographic variables (age and sex). Thus, a total of 110 features were initially included for each sequence-dependent machine learning model. In addition to these uni-sequence models, we also trained multi-sequence models by combining radiomics features from all six sequences along with the same demographic variables.

The machine learning pipeline consisted of two main stages: feature selection and classification. Specifically, four feature selection algorithms—Gain Ratio, Information Gain, ReliefF, and OneR—were used to independently rank the 110 input features based on their relevance to the target outcome (for each sequence and all sequences combined). Each ranked feature list was then paired with one of six classification algorithms: logistic model trees (LMT), random forests (RF), k-nearest neighbors, naïve Bayes, JRip (Repeated Incremental Pruning to Produce Error Reduction), and J48 (a decision tree algorithm based on C4.5), resulting in a comprehensive set of model configurations for both uni- and multi-sequence experiments. These algorithms were used as they have been found to yield good results in previous works (*e.g.* [14–16]*,*).

To identify the optimal subset of features for each configuration, we employed an iterative feature elimination strategy. Starting with the full ranked list, features were removed one at a time in reverse order of importance. At each step, the model was retrained and evaluated using tenfold cross-validation, continuing until only two features remained. The optimal feature subset was defined as the one achieving the highest average classification accuracy and F1-score. To reduce the risk of biased results due to noise or random chance, we also ensured that the best-performing model did not substantially outperform the next-best models without consistent support across folds. In cases of tied performance, the configuration using fewer features was selected in accordance with Occam’s razor [17].

All models were implemented using the WEKA toolkit in the background [18], using default hyperparameter settings to ensure consistency and reduce the risk of overfitting. To prevent information leakage, feature selection and model training were performed exclusively on the training portion of each cross-validation fold, while the corresponding test fold was used solely for evaluation. This nested cross-validation framework ensured strict separation between training and test data at the patient level, thereby preserving sample independence and yielding unbiased performance estimates. No independent dataset was available for external validation.

The complete pipeline (feature selection, model training, and evaluation) was repeated for each of the 20 balanced datasets per classification task.

### Performance metrics

For each task, the accuracy, sensitivity, and the F1-score were calculated [19]. These metrics were computed using the held-out test folds in the tenfold cross-validation framework described above. Thus, final metric values for each classification task reflect averages across all 10 folds and all 20 balanced test datasets, thereby constituting a robustness and sensitivity analysis under the applied class imbalance mitigation strategy.

Feature importance was quantified based on how frequently each feature was included for each task in the optimal subset across the 20 balanced datasets. This frequency served as an indicator of both the stability and relevance of each input feature, highlighting those that contributed most consistently to model performance. In addition to these summary statistics, average confusion matrices were generated to provide a detailed breakdown of class-specific performance for each outcome. The confusion matrices can be found in the Appendix.

### AI reporting standards

This study was conducted and reported in accordance with the 2024 update of the Checklist for Artificial Intelligence in Medical Imaging (CLAIM) [20]. Items were addressed where applicable to the retrospective and exploratory nature of this work, and checklist elements related to external testing and clinical trial registration were considered not applicable and are discussed accordingly. The dataset source, preprocessing steps, reference standards, model development, and internal testing procedures are described in detail to support transparency and reproducibility.

## Results

### WHO CNS grade (II, III, IV)

#### Uni-sequence

For classifying the WHO CNS grade, the best-performing model was based on FLAIR radiomics features, using an LMT classifier with OneR feature selection, achieving a mean accuracy of 57.40% and an F1-score of 57.00%. The models trained on other sequences, including T1-weighted (55.40%), T2-weighted (56.55%), ASL (55.77%), DWI (55.35%), and SWI (56.59%), showed similar but slightly lower levels of performance.

Feature selection revealed consistent reliance on demographic features, with age at imaging selected in all 20 iterations and sex frequently selected across sequences (Fig. 1b). Texture-based features, primarily from GLCM, GLSZM, GLRLM, and GLDM, dominated across sequences, underscoring spatial gray-level heterogeneity as the main discriminative characteristic. Specifically, the DWI and SWI models relied on GLCM-derived measures (*e.g.,* cluster prominence, cluster shade), whereas the T1- and T2-weighted models emphasized texture and shape descriptors capturing tumor morphology. The top performing FLAIR-based model integrated texture, shape, and first-order statistics (*e.g.,* kurtosis, interquartile range), while the ASL-based model relied mainly on shape features (*e.g.,* maximum 2D/3D diameter) with limited texture contribution. Overall, texture heterogeneity remained the most stable and discriminative imaging signature across sequences, complemented by demographic factors.

#### Multi-sequence

The model combining features from all MRI sequences, using an LMT classifier with ReliefF feature selection, achieved a mean accuracy of 56.47% and an F1-score of 56.17%. While competitive, its performance did not surpass that of the best uni-sequence model (FLAIR, 57.40%). Feature selection showed that demographic variables were especially influential, with age selected in all 20 iterations and sex in 14. Among radiomic predictors, DWI contributed most of the frequently selected features, particularly GLSZM texture measures related to zone size and gray-level non-uniformity. FLAIR added important first-order intensity features, such as kurtosis, interquartile range, and entropy. Fewer features were selected from the T1-weighted, T2-weighted, ASL, or SWI sequences, although some occasional contributions were observed. Overall, the multi-sequence model emphasized demographic factors, texture characteristics from DWI, and intensity metrics from FLAIR.

Results are shown in Table 1.

**Table 1:** Performance metrics for WHO CNS Grade, Final Pathological Diagnosis and IDH status(Cross-Validation, Mean ± Standard Deviation)

WHO CNS Grade					
Sequence	Feature Count	Accuracy (%)	F1-score (%)	Recall (%)	Method
ASL	23.15	55.77 ± 4.28	55.19 ± 4.00	55.77 ± 4.00	LMT + ReliefF
DWI	19.85	55.35 ± 3.70	55.17 ± 4.00	55.35 ± 4.00	RF + InfoGain
FLAIR	49.4	**57.40 ± 3.17**	**57.00 ± 3.00**	**57.40 ± 3.00**	LMT + OneR
SWI	16.45	56.59 ± 2.88	56.44 ± 3.00	56.59 ± 3.00	RF + InfoGain
T1	26.9	55.42 ± 3.23	54.90 ± 3.00	55.42 ± 3.00	LMT + ReliefF
T2	44.35	56.55 ± 3.61	56.15 ± 4.00	56.54 ± 4.00	LMT + ReliefF
ALL COMBINED	39.05	56.47 ± 3.76	56.17 ± 4.00	56.47 ± 4.00	LMT + InfoGain
*Final Pathological Diagnosis*					
ASL	41.4	81.50 ± 1.53	81.50 ± 1.00	81.51 ± 1.00	LMT + InfoGain
DWI	28.05	79.42 ± 1.78	79.42 ± 2.00	79.42 ± 2.00	LMT + ReliefF
FLAIR	26.45	80.82 ± 1.80	80.81 ± 2.00	80.81 ± 2.00	LMT + ReliefF
SWI	36.75	81.24 ± 2.04	81.23 ± 2.00	81.24 ± 2.00	LMT + ReliefF
T1	40.95	79.91 ± 1.21	79.91 ± 1.00	79.91 ± 1.00	LMT + ReliefF
T2	25.45	80.60 ± 1.97	80.59 ± 2.00	80.60 ± 2.00	LMT + InfoGain
ALL COMBINED	45.7	80.55 ± 1.37	80.54 ± 1.00	80.55 ± 1.00	LMT + ReliefF
*IDH Status*					
ASL	21.35	62.53 ± 2.86	62.04 ± 3.00	62.52 ± 3.00	LMT + ReliefF
DWI	44.15	62.53 ± 3.51	61.75 ± 3.00	62.53 ± 3.00	JRip + InfoGain
FLAIR	45.45	65.32 ± 3.67	64.81 ± 4.00	65.32 ± 4.00	JRip + ReliefF
SWI	11.65	63.98 ± 3.11	63.49 ± 3.00	63.98 ± 3.00	LMT + ReliefF
T1	32.35	**66.67 ± 2.56**	**66.36 ± 3.00**	**66.65 ± 2.00**	LMT + ReliefF
T2	22.95	63.12 ± 3.74	62.58 ± 4.00	63.12 ± 4.00	LMT + ReliefF
ALL COMBINED	39.4	65.70 ± 3.49	65.49 ± 2.00	65.70 ± 1.00	LMT + ReliefF

### Final pathological diagnosis (glioblastoma vs. astrocytoma)

#### Uni-sequence

For classifying the final pathological diagnosis, the best-performing model was based on ASL-derived imaging features with an LMT classifier and Information Gain feature selection, achieving a mean accuracy and F1-score of 81.50%. The other machine learning models using features derived from standard MRI sequences, including the T1-weighted (79.91%) and T2-weighted MRI sequences (80.60%), and advanced sequences like DWI (79.42%), SWI (81.24%), and FLAIR (80.82%), achieved comparably high performance. Across sequences, performance differences were minor, indicating that most MRI modalities carried sufficient discriminative information for distinguishing glioblastoma from astrocytoma.

Feature selection revealed consistent inclusion of demographic variables, with age at imaging selected in all 20 iterations and sex frequently retained across sequences (Fig. 1a). The ASL-based model, which achieved the highest accuracy, emphasized GLCM-derived texture measures (*e.g.,* autocorrelation, cluster prominence, contrast) and first-order statistics (*e.g.,* total energy, variance, uniformity). Similar texture-driven patterns were observed in the T2-weighted and DWI models, both highlighting GLCM and related features capturing signal and diffusion heterogeneity. In contrast, the T1-weighted and SWI-based models incorporated stronger shape and geometric components (*e.g.,* major-axis length, flatness, elongation), complemented by GLDM, GLRLM, and first-order statistics. The FLAIR-based model exhibited a balanced profile, combining texture, shape, and intensity-based features. Overall, texture and first-order statistics, together with demographic factors, represented the most stable and discriminative predictors of glioblastoma versus astrocytoma.

#### Multi-sequence

The model combining features from all MRI sequences with an LMT classifier and ReliefF feature selection achieved an accuracy of 80.55%, which was comparable to but did not surpass the best-performing uni-sequence models (ASL, 81.50%). Feature selection again highlighted the importance of demographic information, with age consistently retained across all 20 iterations. Among radiomic predictors, T1-weighted imaging dominated the selected features, particularly GLCM and GLRLM descriptors that captured heterogeneity and entropy-related characteristics of the tumor microenvironment. SWI provided complementary information, most notably through first-order intensity features (*e.g.,* maximum, percentiles) and gray-level emphasis measures. Occasional contributions from ASL, FLAIR, DWI, and T2-weighted imaging were observed, but these were less frequent. Overall, the multi-sequence model emphasized age, T1-derived texture and heterogeneity metrics, and SWI-derived intensity and gray-level distribution features.

### IDH status (mutated, wildtype, IDH1)

#### Uni-sequence

For IDH status classification, the best-performing model was based on T1-weighted features with an LMT classifier and ReliefF feature selection, achieving a mean accuracy of 66.66% and an F1-score of 66.36%. The machine learning models using radiomic features derived from FLAIR (65.32%), SWI (63.97%), and other sequences like ASL (62.52%), DWI (62.52%), and T2-weighted (63.11%) showed comparable but slightly lower performance.

Feature selection revealed consistent dominance of shape-based descriptors across all sequences (Fig. 1c), with sphericity, surface-to-volume ratio, and maximum 3D diameter selected in nearly all iterations. The T1-weighted and FLAIR-based models incorporated additional GLSZM and GLCM texture features (*e.g.,* gray-level non-uniformity, correlation, difference entropy) and first-order statistics (*e.g.,* mean, energy), indicating minor contributions from intensity heterogeneity. The DWI-based model showed greater texture involvement, with frequent inclusion of GLCM-derived measures reflecting microstructural heterogeneity relevant to IDH classification. In contrast, the SWI, T2-weighted, and ASL-based models remained predominantly morphology-driven, relying mainly on geometric features with limited texture contributions. Overall, macroscopic tumor geometry, rather than texture or intensity-based heterogeneity, represented the most stable and discriminative imaging signature for IDH status classification.

#### Multi-sequence

The model combining features from all MRI sequences with an LMT classifier and ReliefF feature selection achieved an accuracy of 65.70%, which was comparable to but did not surpass the best-performing uni-sequence model (T1-weighted, 66.56%). Feature selection was dominated by shape descriptors, with sphericity and surface–volume ratio consistently retained across nearly all iterations, along with surface area and multiple axis- and diameter-based metrics. These findings underscore a strong morphological signature associated with IDH status. Beyond shape, additional contributions came from T2-weighted imaging (first-order features including energy, median, and percentiles), SWI (GLSZM gray-level non-uniformity and GLCM/GLRLM texture measures), and T1-weighted imaging (GLCM heterogeneity descriptors such as joint entropy and inverse variance). Occasional features from ASL, FLAIR, and DWI were also selected, but far less consistently. Overall, the multi-sequence model emphasized morphological tumor characteristics, complemented by intensity- and heterogeneity-based predictors from T2, SWI, and T1.

## Discussion

The main finding of this study is that MRI sequence selection can be optimized for specific image-based glioma classification and assessment tasks, balancing diagnostic accuracy and clinical efficiency, across sequences (ASL, DWI, FLAIR, SWI, T1-weighted, T2-weighted), with a combination of ASL, SWI, FLAIR and post-contrast T1-weighted showing the most promising results across all endpoints.

### Clinical implications

For WHO CNS Grade, the machine learning models employing FLAIR imaging emerged as top performing, with an accuracy of 57.40%, leveraging interpretable features like patient age, tumor shape, and first-order intensity metrics. The machine learning models using features derived from advanced MRI sequences, like ASL, DWI, and SWI, showed comparable performance (55.35–56.59% accuracy). However, the lower Grade IV sensitivity for the DWI-based model (62.79%) and the higher Grade III sensitivity of the SWI-based model (49.65%) indicate distinct roles, with SWI’s glcm texture stability potentially adding value despite overlapping features with DWI. Based on the sensitivities, which were derived from the confusion matrices, the same picture can be painted. The highest sensitivities were found for ASL for Grade II, FLAIR for Grade IV and SWI for Grade III tumors. It remains to be investigated why certain image sequences and corresponding contrast mechanisms outperform others in specific glioma classification tasks. Based on previous knowledge about tumor histology and MRI contrast properties, several plausible explanations may be considered. For instance, FLAIR sequences may excel at WHO CNS grade prediction due to their superior sensitivity to peritumoral edema and infiltrative tumor margins, features that are often associated with histological aggressiveness, including increased cellularity and blood–brain barrier disruption [21].

For the final pathological diagnosis (Glioblastoma *vs.* Astrocytoma), interestingly, the machine learning models using imaging features derived from ASL (perfusion imaging) achieved superior performance (81.50% accuracy, 81.50% F1-score), with strong sensitivities for both classes, mainly driven by glcm and first-order features alongside demographics. This highlights the utility of ASL imaging for binary pathological distinction, complementing standard sequences in research or complex cases. The machine learning models using standard sequences (T1-weighted, T2-weighted, and FLAIR) and other functional sequences (DWI, SWI) performed robustly, yet with a lower accuracy (79.43—81.24%). In this binary classification, again the ASL and FLAIR-based models showed the highest sensitivities, reflecting their potential for correct classification between glioblastoma and astrocytoma. However, in this case, the ranges between sequences are comparably low (77.35%-80.35% for astrocytoma and 80.97%-82.70% for glioblastoma), indicating that such a classification can be successfully achieved with each commonly used MRI protocol or combination. In this case, it should also be noted that the sensitivity for ASL to detect glioblastoma is just 0.05% lower than for FLAIR (82.65% ASL vs. 82.70% FLAIR). For astrocytoma, the difference between the ASL (80.35%) and FLAIR-based models (78.94%), on the other hand, is larger, thus suggesting an overall advantage of the perfusion imaging method. ASL may perform best at final pathological diagnosis by quantifying cerebral blood flow as a surrogate for tumor vascularity and neoangiogenesis, thereby capturing perfusion-related differences between histopathological subtypes that are less apparent on structural imaging [22].

For the IDH status, the machine learning model using imaging features derived from T1-weighted MRI excelled (66.66% accuracy, 66.36% F1-score), exhibiting a strong sensitivity for wildtype (71.14%) and moderate performance for mutated and IDH1 gliomas, primarily driven by shape metrics. However, placing these results in context, this accuracy still cannot be seen as sufficient for a standalone diagnosis of IDH. In that scenario, DWI and SWI exhibit a texture overlap, which suggests no additional benefit, emphasizing efficient molecular profiling protocols across all sequences. In this endpoint, combining the sequences showed that the T1-weighted imaging outperformed in both mutated (63.23%) and IDH1 (57.74%) cases while the FLAIR-based model performed best for Wildtype cases (80.65%). Despite being generally lower in the sensitivities, it is an interesting finding that the T1-weighted MRI model shows the highest sensitivities when assessing the mutation status of tumors, which can most likely be attributed to the contrast agent behavior. As the shape metrics in this endpoint were the most selected ones, the sharp edges of the textures accentuated by gadolinium likely contribute to this finding. The T1 weighting may perform best in IDH status prediction by reflecting contrast enhancement patterns related to necrosis and vascular permeability differences between IDH-mutant (non-enhancing) and IDH-wildtype (enhancing) tumors, potentially aligning with underlying molecular histological traits [23].

Importantly, the results from the multi-sequence approach suggest that combinations of sequences do not outperform a single-sequence approach. Thus, for certain classification tasks, even a singular MRI sequence could be considered and suitable for categorization of the pathology. While this is a theoretical possibility that could save time and make the imaging process more efficient, in clinical practice, a multi-sequence approach benefits from being safer for stating any diagnosis as it incorporates an additional viewpoint and a different (patho-)physiological perspective of the patient. These examples include DWI being fast to acquire (often under one minute) to measure diffusion restrictions in the tumor compartment; the use of SWI to visualize microhemorrhages and vessels within the tumor; and T2w imaging to assess the T2/FLAIR mismatch, an indicator of IDH-mutated astrocytomas [24]. As these are well-known and established radiological signs, their clinical value remains significant in routine practice. However, with the advances in automation, it remains to be evaluated whether such methods might influence future sequence prioritization.

In principle, integrating the presented post-processing tools into PACS systems could automate radiomics feature extraction and classification, enabling real-time decision-support during tumor boards or treatment planning, potentially enhancing patient outcomes in glioma management. Nevertheless, the presented models should be regarded as exploratory and hypothesis-generating at this point rather than immediately clinically deployable diagnostic or decision-support tools. External validation in independent, multi-institutional cohorts will be necessary before consideration of routine clinical implementation.

While the classification performance across the three tasks was promising, no single machine learning configuration consistently outperformed the others. This underscores the dependence of model performance on the specific combination of classifier and feature selection strategy, and suggests that task-adapted pipelines may be more appropriate than a uniform, one-size-fits-all approach for radiomics-based glioma classification. For instance, setups using ReliefF feature selection tended to prioritize shape-based features, whereas information gain emphasized entropy-reducing texture and intensity features, differences that may partly explain the task-specific performance of sequences like ASL and T1-weighted MRI. Moreover, the variability in selected features across model configurations highlights the challenge of identifying stable, clinically interpretable radiomic signatures. Taken together, the observed performance patterns indicate that classical radiomics features capture task-specific, sequence-dependent information. While the discriminative capacity for multi-class endpoints, such as WHO CNS grade and IDH status, remains limited, stronger separability was observed for the glioblastoma *vs.* astrocytoma distinction.

This study has several limitations. First, all experiments were conducted using a single-institution dataset (UCSF-PDGM), which may limit the generalizability of the results to broader patient populations or imaging protocols, as radiomics signatures can be prone to acquisition variability. Thus, external validation across multi-institutional cohorts will be essential to determine the robustness and broader applicability of the observed findings. Second, although feature selection was performed within a nested cross-validation framework to reduce bias, the high dimensionality of radiomic features means that there is still potential for selection-related variability. Third, the IDH1 and IDH2 subtypes were modeled separately, deviating from the conventional clinical categorization of IDH-mutant vs. IDH-wildtype tumors. Although this enabled exploration of potential subtype-specific radiomic patterns, it may limit direct clinical interpretability. Lastly, deep learning-based methods were not evaluated in this study. While this is a limitation given their increasing prominence in neuro-oncologic imaging, our use of classical machine learning models provided multiple advantages with respect to handling tabular radiomic data, mitigating overfitting in a moderate-sized cohort, and maintaining interpretability. Nevertheless, deep learning models may capture complementary imaging representations and achieve higher predictive performance so that the reported findings should be interpreted as reflecting the performance of a traditional radiomics pipeline rather than the upper bound of achievable accuracy. Future studies should directly compare classical radiomics, deep learning, and hybrid approaches, ideally using larger, multi-institutional datasets where more complex modeling may be advantageous.

## Conclusion

This work examined the usability of an exemplary comprehensive MRI protocol to evaluate its use in the differential diagnosis of glioma. The results of this work suggest that only a subset of sequences is needed to maximize the information gained. For WHO CNS Grade, machine learning models based on FLAIR offer the highest accuracy (57.40%), while for the final pathological diagnosis, ASL-based models achieve the best accuracy (81.50%), and finally, for IDH status, machine learning models based on T1-weighted perform optimally (66.66%).

## Supplementary Information

Below is the link to the electronic supplementary material.

Supplementary file1 (DOCX 608 KB)
