# Supplementary material for: Optimizing MRI sequence selection for glioma classification: a radiomics-based analysis of WHO CNS grade, final pathological diagnosis, and IDH status
Source: Radiol Med. 2026 Sep 6;131(10):1877–85. doi: 10.1007/s11547-026-02218-9 (PMC13668893; doi:10.1007/s11547-026-02218-9)
Supplement: Supplementary file 1 [file 11547_2026_2218_MOESM1_ESM.docx]

**Appendix:**

1. **Calculation of the overall F-Score**

The F1 score is the harmonic mean of precision and recall (sensitivity), providing a balanced measure of a classifier's performance. For a single class, the F1 score is calculated as:

$${F1}_{c}=2x\frac{{Precision}_{c} x {Recall}_{c}}{{Precision}_{c}+{Recall}_{c}}$$

where:

$$Precision= \frac{{TP}_{c}}{{TP}_{c}+{FP}_{c}}$$

$$Recall= \frac{{TP}_{c}}{{TP}_{c}+{FN}_{c}}$$

with FP_c_ being the false positives (sum of column c excluding the diagonal) and FN_c_ being the false negatives (sum of row c excluding the diagonal).

For the multi-class problems, the overall F1 score is the macro-averaged F1 score, computed by:

1. Calculating the F1 score for each class individually using the confusion matrix
2. Averaging the per-class F1 scores across all classes

For the binary classification, the overall F1 score is calculated as:

1. The macro-averaged F1 score across both classes (Glioblastoma and Astrocytoma).

The finally resulting values were corroborated by computing per-class F1 scores from the confusion matrices and averaging them.

2. **Confusion Matrices**

**ASL**

IDH Status

| True \ Predicted | Mutated | Wildtype | IDH1 |
| --- | --- | --- | --- |
| Mutated | 17.85 | 4.75 | 8.4 |
| Wildtype | 3.4 | 24.4 | 3.2 |
| IDH1 | 10.05 | 5.05 | 15.9 |

WHO CNS Grade

| True \ Predicted | Grade II | Grade III | Grade IV |
| --- | --- | --- | --- |
| Grade II | 30.15 | 7.25 | 5.6 |
| Grade III | 10.6 | 17.85 | 14.55 |
| Grade IV | 8.15 | 10.9 | 23.95 |

Pathological Diagnosis

| True \ Predicted | Glioblastoma | Astrocytoma |
| --- | --- | --- |
| Glioblastoma | 93.4 | 19.6 |
| Astrocytoma | 22.2 | 90.8 |

**DWI**

IDH Status

| True \ Predicted | Mutated | Wildtype | IDH1 |
| --- | --- | --- | --- |
| Mutated | 18.95 | 5.45 | 6.6 |
| Wildtype | 1.9 | 24.75 | 4.35 |
| IDH1 | 10.85 | 5.7 | 14.45 |

WHO CNS Grade

| True \ Predicted | Grade II | Grade III | Grade IV |
| --- | --- | --- | --- |
| Grade II | 27 | 8.8 | 7.2 |
| Grade III | 11.15 | 19.2 | 12.65 |
| Grade IV | 6.1 | 11.7 | 25.2 |

Pathological Diagnosis

| True \ Predicted | Glioblastoma | Astrocytoma |
| --- | --- | --- |
| Glioblastoma | 92.1 | 20.9 |
| Astrocytoma | 25.6 | 87.4 |

**FLAIR**

IDH Status

| True \ Predicted | Mutated | Wildtype | IDH1 |
| --- | --- | --- | --- |
| Mutated | 18.85 | 4.7 | 7.45 |
| Wildtype | 2.55 | 25 | 3.45 |
| IDH1 | 8.65 | 5.45 | 16.9 |

WHO CNS Grade

| True \ Predicted | Grade II | Grade III | Grade IV |
| --- | --- | --- | --- |
| Grade II | 28.8 | 8.25 | 5.95 |
| Grade III | 10.95 | 18.65 | 13.4 |
| Grade IV | 6.45 | 9.95 | 26.6 |

Pathological Diagnosis

| True \ Predicted | Glioblastoma | Astrocytoma |
| --- | --- | --- |
| Glioblastoma | 93.45 | 19.55 |
| Astrocytoma | 23.8 | 89.2 |

**SWI**

IDH Status

| True \ Predicted | Mutated | Wildtype | IDH1 |
| --- | --- | --- | --- |
| Mutated | 19.3 | 4.35 | 7.35 |
| Wildtype | 2.75 | 24.05 | 4.2 |
| IDH1 | 8.55 | 6.3 | 16.15 |

WHO CNS Grade

| True \ Predicted | Grade II | Grade III | Grade IV |
| --- | --- | --- | --- |
| Grade II | 28.5 | 8.55 | 5.95 |
| Grade III | 8.8 | 21.35 | 12.85 |
| Grade IV | 8.15 | 11.7 | 23.15 |

Pathological Diagnosis

| True \ Predicted | Glioblastoma | Astrocytoma |
| --- | --- | --- |
| Glioblastoma | 93.1 | 19.9 |
| Astrocytoma | 22.5 | 90.5 |

**T1**

IDH Status

| True \ Predicted | Mutated | Wildtype | IDH1 |
| --- | --- | --- | --- |
| Mutated | 19.6 | 4.65 | 6.75 |
| Wildtype | 3.3 | 24.5 | 3.2 |
| IDH1 | 7.75 | 5.35 | 17.9 |

WHO CNS Grade

| True \ Predicted | Grade II | Grade III | Grade IV |
| --- | --- | --- | --- |
| Grade II | 29.05 | 8.55 | 5.4 |
| Grade III | 12.25 | 17.25 | 13.5 |
| Grade IV | 5.8 | 12 | 25.2 |

Pathological Diagnosis

| True \ Predicted | Glioblastoma | Astrocytoma |
| --- | --- | --- |
| Glioblastoma | 91.5 | 21.5 |
| Astrocytoma | 23.9 | 89.1 |

**T2**

IDH Status

| True \ Predicted | Mutated | Wildtype | IDH1 |
| --- | --- | --- | --- |
| Mutated | 18 | 5.65 | 7.35 |
| Wildtype | 2.65 | 24.1 | 4.25 |
| IDH1 | 9.3 | 5.1 | 16.6 |

WHO CNS Grade

| True \ Predicted | Grade II | Grade III | Grade IV |
| --- | --- | --- | --- |
| Grade II | 29.55 | 9 | 4.45 |
| Grade III | 10.2 | 18.2 | 14.6 |
| Grade IV | 7.25 | 10.55 | 25.2 |

Pathological Diagnosis

| True \ Predicted | Glioblastoma | Astrocytoma |
| --- | --- | --- |
| Glioblastoma | 92.95 | 20.05 |
| Astrocytoma | 23.8 | 89.2 |

Supplemental Figures:

Figure 1: Frequency of selected radiomics features of all included MRI sequences for Final Pathological Diagnosis

Figure 2: Frequency of selected radiomics features of all included MRI sequences for WHO CNS Grade

Figure 3: Frequency of selected radiomics features of all included MRI sequences for IDH status
